# Associations between Korean Adolescents’ Sexual Orientation and Suicidal Ideation, Plans, Attempts, and Medically Serious Attempts

**Published:** 2017-04

**Authors:** Yeunhee KWAK, Ji-Su KIM

**Affiliations:** Faculty of Red Cross College of Nursing, Chung-Ang University, Seoul, Republic of Korea

**Keywords:** Adolescents, Bisexual, Gay, Heterosexual, Homosexual, Suicide, Korea

## Abstract

**Background:**

Despite growing interest in the public health of sexual minority, youth around the world due to the high rates of suicidal ideation and attempts in this population, few studies on the sexual orientation of Korean adolescents have been conducted. Therefore, this study investigated the relationship between the sexual orientation of Korean adolescents and their suicide-related behavior.

**Methods:**

Raw data from the tenth Korea Youth Risk Behavior Web-based Survey were analyzed by logistic regression analysis. The sample consisted of 3603 adolescents who provided selected demographic variables and reported on their experience of sexual intercourse with the same or the opposite sex, along with lifestyle and suicide-related behaviors.

**Results:**

Rates of suicidal ideation, plans, attempts, and medically serious attempts were higher in both homosexual and bisexual than heterosexual groups. Suicidal ideation (odds ratio 95% confidence interval: 1.09–2.08), suicidal plans (odds ratio 95% confidence interval: 1.01–2.09), and suicide attempts (odds ratio 95% confidence interval: 1.28–2.88) had the strongest associations with homosexuality after multivariate adjustment. In contrast, bisexuality was only significantly associated with suicidal attempts (odds ratio 95% confidence interval: 1.01–2.97) after multivariate adjustment.

**Conclusion:**

Effective suicide prevention interventions are required for homosexual and bisexual adolescents, in the form of targeted programs to improve their mental health status and ability to cope with stress.

## Introduction

The world sees the sexual minority as a population group; thus, the health paradigm of this group has been changed to reflect their own recognition perspective. In Korea, however, the human rights and health of the sexual minority have not yet been discussed in depth. The population of the sexual minority (1), around 3.5% of the adult population of United States, or 8 million people, are lesbian, gay, or bisexual (LGB). Because Korean society is not accepting of the sexual minority, some people find it difficult to identify as a member of this group and it is hard to establish accurate population numbers (2). In Korea, there is around 1 to 5 million in the sexual minority (3), although it is impossible to get accurate statistics (4). Despite this, according to the World Values Survey (2005–2009) (5), Korea had the second-to-lowest mean score (after Turkey) among 17 Organization for Economic Cooperation and Development countries when asked the question “Do you think that homosexuality can be justified?” and was found, as a country, to be greatly hostile toward homosexuality. Adolescence is an important development period during which teenagers experience physical sexual changes and establish their gender identity (6).

LGB adolescents are known to experience confusion, indecision, and uncertainty about whether they are heterosexual, bisexual, or homosexual. In Korean society, homosexuality (the so-called “wrong identity”) is dismissed or ignored among youth as a temporary phenomenon that occurs before the development of heterosexuality (the so-called “right identity”). Although exact population statistics cannot be determined, 7.5% of adolescents in Korea have reported experiencing problems with their gender identity (7, 8).

When individuals are young, and they recognize their sexual identity for the first time, they could face psychological danger due to an increased risk of social prejudice, discrimination, and a decreased ability to address both (9, 10). In addition to typical daily stress, homosexuals are facing a particular stress, called homosexuality-related stress, caused by existing in a society focused on heterosexual love (7, 11). Homosexuals are commonly exposed to stress related to social disgrace or disadvantage. Consequently, they are more likely to perform deviant behaviors such as smoking, drinking, drug use, etc. to escape from the stress; however, these activities have negative effects on their health (8, 12–14).

The emotional pain of grade was examined 9–12 students revealed that homosexual youths were more likely to suffer from depression, self-injury, and suicidal ideation than their heterosexual counterparts (15). According to the 2011 “national school climate study” that included 8584 youth sexual minorities, 81.9% experienced unfair harassment due to their sexual orientation, and 63.5% recognized their school as an unsafe place and did not go to school (16). This negative view, coupled with bullying, and physical/verbal violence were factors that caused suicide (17–19). However, students supported by their families had more positive health results (e.g., self-respect) and they could better protect themselves from negative health results (e.g., depression, suicidal compulsion) (12, 20).

Around the world, LGB people in adolescence or early adulthood experience bullying (15, 21–23) because of heterosexuality-centered cultural norms and habituated homophobia (24). As a result, teenaged LGB people experience more severe stress and poorer mental health status than their heterosexual peers (25–27). In particular, as sexual minority youth (SMY) are more likely to experience exclusion from their family or acquaintances, and have higher rates of depression, illegal drug consumption, and infection with acquired immune deficiency syndrome or other sexual diseases (12). SMY have been reported to have a higher rate of suicidal ideation and suicide attempts than the general population (17, 28–30). According to previous studies using small convenience samples (31, 32), rates of suicidal ideation and suicide attempts among SMY reached 20%–40%, which is 5–6 times higher than those of non-SMY (33–35). However, less is known about the full range of suicide-related behaviors, including suicidal ideation and plans, and suicide attempts among SMY (30). Knowing about the prevalence of these outcomes is crucial, as they indicate the likelihood of sustained injuries and risks for future suicide, suicide attempts, or repeated attempts (36).

The process of “coming out” to one’s family for the first time is critical to the sexual identity and a great factor of stress for homosexual youths (11). The experience of being excluded from or being unfairly treated by family, friends, and classmates causes psychological stress to Korean youth homosexuals and results in suicidal ideation (7). Moreover, it causes youth sexual minorities to have low social and psychological self-esteem and high depression levels when compared to other student groups (19, 37).

Despite increasing interest in the LGB population in connection with diverse public health-related problems around the world, there is a lack of research on the sexual orientation of youth in Korea, given the conservative views wherein sex is commonly regarded as taboo. This means that the Korean government lacks information that is applicable to the public health concern of SMY (38). Therefore, this study investigated the relationship between the Korean adolescents’ sexual orientation and suicide-related behavior to fill this gap in the literature.

The specific purposes of this study included the following: 1) to identify the associations between demographic characteristics and sexual orientation, and 2) to identify the associations between adolescents’ sexual orientation and suicidal ideation, plans, attempts, and medically serious attempts.

## Materials and Methods

### Design and sample

We used a cross-sectional study design to identify the associations between sexual orientation and suicidal ideation, plans, attempts, and medically serious attempts among adolescents in Korea. Raw data were sourced from the tenth Korea Youth Risk Behavior Web-based Survey (KYRBWS-X), conducted by the Korea Centers for Disease Control and Prevention (KCDC). The KYRBWS is an anonymous, self-administered online survey conducted to identify the health behaviors of Korean adolescents (middle school freshman to high school seniors) using a complex sample design involving stratification, clustering, and multistage sampling methods. In the KYRBWS-X, 75149 people from 800 schools (400 middle schools and 400 high schools) were surveyed and 74167 (97.2%) people from these 800 schools returned valid responses (1). There were 15319 adolescents experienced sexual intercourse with a member of the same or the opposite sex, and 3603 of them were analyzed in this study.

### Ethical considerations

The KYRBWS is a statistical survey approved by the Korean government (Approval No. 11758), and which received institutional review board deliberation from the KCDC (2014-06EXP-02-P-A).

We requested permission from the KCDC to the use the KYRBWS survey results for research purposes, and submitted a data use plan and posted a written pledge of our intentions on the KYRBWS homepage (http://yhs.cdc.go.kr). The study follows the ethical standards of the Helsinki Declaration, as revised in 2013.

### Study variables and measures

#### Sexual orientation

Sexual orientation describes the tendency to be attracted emotionally and sexually to men, women, or both sexes (39). This study categorized sexual orientation into heterosexual (heterosexual relations experienced), homosexual (homosexual relations experienced), and bisexual (heterosexual and homosexual relations experienced).

### Suicidal ideation, plans, attempts, and medically serious attempts

Suicidal ideation was assessed using the question “Have you ever thought about suicide seriously over the last 12 months?”; suicidal plans were assessed with the question “Have you ever planned suicide in detail over the last 12 months?”; suicide attempts were assessed with the question “Have you ever made a suicide attempt over the last 12 months?”; and medically serious suicide attempts were assessed among youth attempted suicide over the last 12 months using the question “Have you ever had medical treatment in hospital for a suicide attempt?”

### Demographic characteristics

We assessed the following demographic characteristics: age, gender, body mass index (BMI), school level (middle school, high school, or vocational high school), area and size of residence (large city, medium-sized city, or country area), economic status (very high, high, moderate, low, or very low), school performance (very high, high, moderate, low, or very low), subjective health status (very good, good, moderate, poor, or very poor), subjective happiness status (“How happy are you in daily life?,” very happy, happy, moderate, unhappy, and or unhappy), sleep satisfaction (“Did you sleep enough in the last week?,” very satisfied, satisfied, moderately satisfied, dissatisfied, or very dissatisfied), awareness of depression (“Have you ever felt sadness or frustration strong enough that it stopped your daily life for 2 wk during the last year?”) and lifetime experience of drinking, smoking, and drug use.

### Statistical analysis

Statistical analysis was performed using SPSS Complex Sample, version 19.0 (SPSS Inc., Chicago, IL, USA) in a manner that reflected sampling weights and provided nationally representative estimates according to KCDC guidelines. Continuous variables (subjects’ general characteristics) are presented as mean (SE) values, whereas categorical variables are presented as percentage (SE) values. Analyses of variance and chi-square tests were used for comparison of demographic characteristics by sexual orientation. Logistic regression analyses were performed to examine the associations between sexual orientation and suicidal ideation, plans, attempts, and medically serious attempts. Odds ratios (OR) and confidence intervals (CI) were estimated after adjusting for the individual characteristics of age, gender, BMI, and perceived economic status in Model 2, and we then added the health risk behaviors of lifetime drinking experience, lifetime smoking experience, and lifetime drug use in Model 3. A *P*-value under .05 was considered statistically significant.

## Results

Differences in Korean adolescents’ demographic characteristics according to sexual orientation are shown in Table 1. Age was less strongly associated with bisexuality than with heterosexuality (*P*<0.001). There was a high rate of heterosexuality compared to homosexuality or bisexuality across all school levels (*P*<0.001).

**Table 1:** Associations between demographic characteristics and sexual orientation (n = 3603)

**Variable**	**Classification**	**n (%) or Mean ± SE**	**Heterosexual (n=2930)**	**Homosexual (n=393)**	**Bisexual (n=280)**	***P***
			% (SE) or Mean ± SE	% (SE) or Mean ± SE	% (SE) or Mean ± SE	
Age (yr)		15.83 ± 0.03	15.91 ±0.04	15.47 ± 0.10	15.31 ± 0.12	<.001
Body mass index	(kg/m^2^)	20.98 ± 0.05	21.04 ± 0.06	20.43 ± 0.20	20.75 ± 0.22	.011
Gender (%)	Male	2494 (71.5)	72.1 (1.3)	67.5 (2.7)	71.0 (2.8)	.190
	Female	1109 (28.5)	27.9 (1.3)	32.5 (2.7)	29.0 (2.8)	
School type (%)	Middle school	1159 (30.0)	27.0 (1.1)	42.7 (2.8)	44.5 (3.3)	<.001
	High school	1809 (50.0)	51.2 (1.4)	43.5 (2.9)	46.4 (3.4)	
	Vocational high school	635 (20.0)	21.8 (1.3)	13.9 (1.9)	9.1 (1.9)	
Urban scale (%)	Big cities	1518 (41.2)	41.2 (1.4)	37.8 (2.7)	46.1 (3.3)	.364
	Medium sized cities	1794 (52.7)	52.6 (1.5)	56.3 (2.9)	48.3 (3.4)	
	Country area	291 (6.1)	6.2 (0.8)	5.9 (1.5)	5.6 (1.6)	
Economic status	Very high	490 (13.9)	11.4 (0.6)	21.7 (2.1)	30.5 (2.6)	<.001
(%)	High	792 (22.0)	23.2 (0.8)	16.5 (1.8)	16.0 (2.2)	
	Moderate	1333 (36.6)	38.9 (0.9)	26.7 (2.1)	26.3 (2.7)	
	Low	623 (17.1)	17.5 (0.7)	17.8 (1.9)	12.4 (1.9)	
	Very low	365 (10.4)	9.1 (0.6)	17.2 (2.0)	14.8 (2.0)	
School	Very high	522 (14.8)	11.9 (0.6)	23.4 (2.1)	34.2 (3.1)	<.001
Performance	High	641 (17.5)	18.1 (0.8)	15.6 (1.7)	13.8 (2.1)	
(%)	Moderate	834 (22.7)	24.2 (0.8)	19.3 (2.1)	11.8 (2.0)	
	Low	877 (24.4)	25.5 (0.9)	20.5 (2.1)	18.2 (2.3)	
	Very low	729 (20.5)	20.3 (0.7)	21.2 (1.9)	22.0 (2.4)	
Subjective	Very healthy	1085 (30.6)	29.5 (0.9)	32.5 (2.4)	11.7 (1.0)	<.001
Health status (%)	Healthy	1390 (39.0)	40.3 (0.9)	34.4 (2.4)	9.7 (0.8)	
	Moderate	767 (21.0)	21.6 (0.8)	20.6 (2.1)	10.8 (1.2)	
	Poor	295 (7.7)	7.6 (0.5)	8.7 (1.4)	12.4 (1.8)	
	Very poor	66 (1.7)	1.0 (0.2)	3.8 (0.9)	24.4 (5.2)	
Subjective	Very happy	843 (23.5)	22.5 (0.8)	24.9 (2.3)	31.8 (2.8)	<.001
Happiness (%)	Happy	1208 (34.0)	35.2 (0.9)	28.7 (2.5)	28.0 (2.6)	
	Moderate	1030 (28.5)	28.8 (0.9)	30.5 (2.3)	21.8 (2.5)	
	Unhappy	370 (10.0)	10.5 (0.5)	8.7 (1.5)	6.3 (1.4)	
	Very unhappy	152 (4.1)	3.0 (0.3)	7.1 (1.2)	12.2 (2.0)	
Sleep	Very satisfaction	293 (8.1)	7.0 (0.5)	12.7 (1.7)	13.6 (1.9)	<.001
Satisfaction (%)	Satisfaction	540 (15.1)	15.1 (0.6)	15.6 (1.9)	14.8 (2.2)	
	Moderate	998 (27.9)	28.9 (0.8)	26.5 (2.2)	19.1 (2.2)	
	Dissatisfaction	1015 (28.0)	29.0 (0.8)	22.7 (2.0)	25.7 (2.7)	
	Very dissatisfaction	757 (20.9)	20.1 (0.7)	22.4 (2.3)	26.9 (2.7)	
Depression	Yes	1562 (42.7)	41.3 (0.9)	49.2 (2.6)	48.7 (2.9)	.001
Awareness (%)	No	2041 (57.3)	58.7 (0.9)	50.8 (2.6)	51.3 (2.9)	
Lifetime drinking	Yes	2652 (74.3)	76.5 (0.8)	65.0 (2.4)	64.7 (2.8)	<.001
Experience (%)	No	951 (25.7)	23.5 (0.8)	35.0 (2.4)	35.3 (2.8)	
Lifetime smoking	Yes	2097 (59.5)	60.6 (1.1)	51.9 (2.8)	59.2 (3.1)	.007
Experience (%)	No	1506 (40.5)	39.4 (1.1)	48.1 (2.8)	40.8 (3.1)	
Lifetime drug	Yes	333 (9.3)	4.7 (0.5)	32.0 (2.4)	26.6 (2.7)	<.001
Experience (%)	No	3270 (90.7)	95.3 (0.5)	68.0 (2.4)	73.4 (2.7)	

**Fig. 1:**
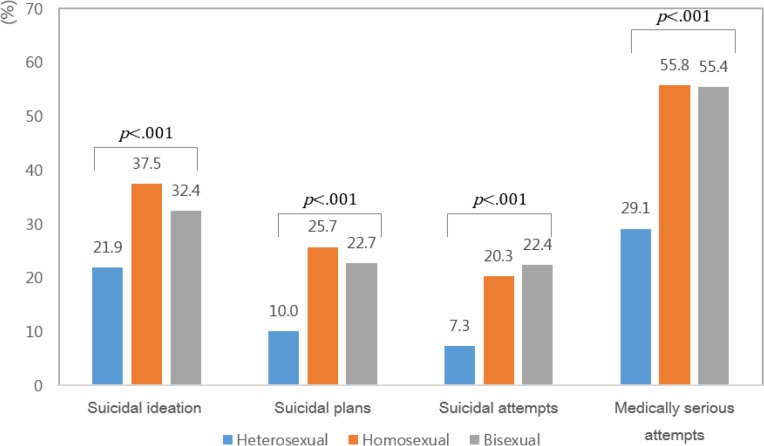
Associations between adolescents’ sexual orientation and suicidal ideation, plans, attempts, and medically serious attempts

A higher economic status was associated with a higher rate of bisexuality, and a lower economic status was associated with a higher rate of homosexuality (*P*<0.001). Students with either low or very high school performance had the highest rate for bisexuality, followed by homosexuality and then heterosexuality (*P*<0.001). Few bisexual respondents reported having a good subjective health status and many reported that they had poor health (*P*<0.001). The rate of respondents with a very unhappy subjective happiness status was the highest in the bisexual group, followed by homosexual and then heterosexual groups (*P*<0.001). Lower satisfaction with sleep was most prevalent in the bisexual group (*P*<0.001). Regarding awareness of depression, the homosexual group had the highest rate, followed by the bisexual and then heterosexual groups (*P*<0.001). The heterosexual group had the highest rate for lifetime experience of drinking and smoking. Regarding drug use, the homosexual group had the highest rate, followed by the bisexual and then heterosexual groups (*P*<0.001). Differences in suicidal ideation, plans, attempts, and medically serious attempts depending on the respondents’ sexual orientation are presented in Fig. 1.

The homosexual group had the highest rate of suicidal ideation, plans, and medically serious attempts, followed by the bisexual and then heterosexual groups (*P*<0.001). In terms of suicide attempts, the bisexual group had the highest rate, followed by the homosexual and then heterosexual groups (*P*<0.001). The associations between adolescents’ sexual orientation and suicidal ideation, plans, attempts, and medically serious attempts are shown in Table 2.

**Table 2:** Associations between adolescents’ sexual orientation and suicidal ideation, plans, attempts, and medically serious attempts (n = 3603)

	**Suicidal ideation OR (95% CI)**	**Suicidal plans OR (95% CI)**	**Suicidal attempts OR (95% CI)**	**Medically serious attempts OR (95% CI)**
Model 1				
Heterosexual	1	1	1	1
Homosexual	2.14 (1.69–2.69)	3.13 (2.48–3.96)	3.21 (2.43–4.23)	3.08 (1.80–5.27)
Bisexual	1.70 (1.30–2.24)	2.65 (1.92–3.67)	3.64 (2.63–5.04)	3.03 (1.82–5.06)
Model 2				
Heterosexual	1	1	1	1
Homosexual	1.58 (1.17–2.14)	1.77 (1.26–2.47)	2.23 (1.52–3.29)	2.63 (1.30–5.30)
Bisexual	1.38 (0.99–1.93)	1.41 (0.89–2.23)	1.97 (1.21–3.19)	1.46 (0.59–3.62)
Model 3				
Heterosexual	1	1	1	1
Homosexual	1.52 (1.09–2.08)	1.45 (1.01–2.09)	1.92 (1.28–2.88)	1.39 (0.64–3.03)
Bisexual	1.34 (0.95–1.89)	1.21 (0.72–2.00)	1.73 (1.01–2.97)	1.36 (0.52–3.51)

Model 1: univariate; Model 2: adjusted for age, gender, BMI and perceived economic status; Model 3; Model 2 and adjusted lifetime drinking experience, lifetime smoking experience and lifetime drug experience

The unadjusted logistic regression analyses revealed that the odds of suicidal ideation, plans, attempts, and medically serious attempts were significantly associated with sexual orientation. In Model 2, where we adjusted for age, gender, BMI, and economic status, the odds of suicidal ideation, plans, attempts, and medically serious attempts were significantly associated with sexual orientation. In Model 3, where we adjusted for age, gender, BMI, economic status, and lifetime experience of drinking, smoking, and drug use, the odds of suicidal ideation, plans, and attempts were significantly associated with sexual orientation. More specifically, the unadjusted analysis showed that when the heterosexual group was set as the reference, the OR for suicidal ideation was 2.14 in the homosexual group and 1.70 in the bisexual group; the OR for suicidal plans was 3.13 in the homosexual group and 2.65 in the bisexual group; the OR for suicide attempts was 3.21 in the homosexual group and 3.64 in the bisexual group; and the OR for medically serious suicide attempts was 3.08 in the homosexual group and 3.03 in the bisexual group. In Model 2, where the respondents’ age, gender, BMI, and economic status were adjusted, when the heterosexual group was set as the reference. The OR for suicidal ideation was 1.58 in the homosexual group; the OR for suicidal plans was 1.77 in the homosexual group; the OR for suicide attempts was 2.23 in the homosexual group and 1.97 in the bisexual group, and the OR for medically serious suicide attempts was 2.63 in the homosexual group. In Model 3, where the respondents’ general characteristics and their health risk behaviors of lifetime experiences of drinking, smoking, and drug use were adjusted, when the heterosexual group was set as the reference. The OR of suicidal ideation was 1.52 in the homosexual group; the OR of suicidal plans was 1.45 in the homosexual group, and the OR of suicide attempts was 1.92 in the homosexual group and 1.73 in the bisexual group.

## Discussion

In this study, Korean adolescents’ sexual orientation is associated with suicidal ideation, plans, attempts, and medically serious attempts. These results provide fundamental support for the need to improve the mental health and public health concern among Korean SMY.

Korean adolescents with varying sexual orientations showed statistically significant differences in the demographic characteristics of age, school level, economic status, school performance, subjective health status, subjective happiness status, sleep satisfaction, awareness of depression, and lifetime experience of drinking and smoking. Homosexual and bisexual groups had poorer general and mental health status (9,18,40,41), higher rates of smoking and alcohol use (8,13), and lower economic status (10) than the heterosexual group did. In particular, LGB individuals were economically isolated and faced homophobia and discrimination every day, causing them to experience severe stress and adopt negative lifestyle choices, such as smoking and drinking (8,14). Of the assessed health risk behaviors, tobacco and alcohol use as related to morbidity and mortality are critical public health problems (14,42). Accordingly, aside from the non-smoking and drinking programs created for the general population, it is necessary to develop campaigns and programs targeted at SMY to reduce smoking and excessive drinking in this group.

According to the analysis of differences in suicidal ideation, plans, attempts, and medically serious attempts depending on the subjects’ sexual orientation, the homosexual group had the highest rates of suicidal ideation and plans, and medically serious suicide attempts and the bisexual group had the highest rate of suicide attempts. Given that adolescents’ sexual orientation was found to be significantly associated with suicidal ideation, plans, attempts, and medically serious attempts, there were differences between the groups depending on the adjusted variables. Nevertheless, when the heterosexual group was set as the reference, the OR of variables related to suicide was largest in the heterosexual group, followed by the homosexual group and then the bisexual group. On balance, adolescents with a homosexual or bisexual orientation had the highest risk for suicide-related variables and the poorest mental health status. “LGB, compared to heterosexual, people are at higher risk of developing mental health disorders, suicidal ideation, substance misuse, and deliberate self-harm” (43).

According to the 2013 South Korean Lesbian, Gay, Bisexual, Transgender and Intersex Community Social Needs Assessment Survey, suicide attempts among SMY reached 46%, and the frequency of deliberate self-harm was 53% (44). These figures are much higher than the 2011 general population rate of Korean adolescents’ suicide attempts (4.4%) (45). Further, the elevated odds of medically serious suicide attempts among homosexual and bisexual groups in this study indicate this is perhaps the greatest risk indicator of future suicide considering the potential associated intent (46, 47). The suicide-related behaviors of Korean SMY must be taken seriously; however, there is currently no forum for SMY to ask for help, and it is difficult to prevent self-harm, suicide, or other fatal circumstances in this group. In this sense, there is a need to raise awareness of the necessity of public policy to address issues related to suicide among SMY and enhance protection of and support for this group. LGB individuals have been found to experience more conflicts with acquaintances who reject the sexual minority gender identity, and this causes them more distress than does their sexual orientation or gender identity (7). “A hostile social environment characterized by stigma, prejudice, and discrimination may be associated with increases in individual risk factors for suicide, including depression, substance abuse, social isolation, peer conflict, and victimization” (18, 43, 48). The physical and psychological problems of adolescents who are immature, sensitive, and have yet to establish a gender identity should be of primary public concern in Korea. Therefore, we consider that the constant interest in and support for vulnerable populations as SMY will reduce their suicide-related behaviors.

This study has some limitations. First, as it was cross-sectional in design, we cannot determine the causal relationships between sexual orientation and suicidal ideation, plans, attempts, and medically serious attempts among Korean adolescents. Second, the data were collected using an anonymous, self-administered online survey; thus, some participants lied about their sexual orientation. However, the anonymous nature of the survey should reduce the likelihood of social desirability bias compared to other data collection methods, such as face-to-face interview. Moreover, because the values and prejudices towards sexuality vary by country, it is difficult to generalize the results of this study to different countries. Despite these limitations, this study has some noteworthy strength. To the best of our knowledge, this is the first study to use a large, nationally representative sample to examine sexual orientation and suicide-related behaviors among Korean adolescents. Moreover, we adjusted our analyses for many covariates to minimize their potential influence.

## Conclusion

SMY had a higher risk of suicide-related behaviors than heterosexual youth did, especially concerning medically serious suicide attempts. Therefore, counseling or educational programs be developed to prevent suicide and suicide attempts among SMY. In addition, it is necessary to implement public policy to control mental health problems related to adolescents’ sexual orientation or gender identity.

## Ethical considerations

Ethical issues (Including plagiarism, informed consent, misconduct, data fabrication and/or falsification, double publication and/or submission, redundancy, etc.) have been completely observed by the authors.
